# Cell-in-Cell Structures in Colorectal Cancer: A Proposed Assessment Method and Correlation with Established Poor Prognostic Factors

**DOI:** 10.3390/jpm15120591

**Published:** 2025-12-03

**Authors:** Arseniy Potapov, Ruslan Spashchanskii, Aleksey Kazakov, Anastasiya Shepeleva, Uliana Lisitsa, Marina Bugrova, Irina Druzhkova

**Affiliations:** 1 Institute of Experimental Oncology and Biomedical Technologies, Privolzhsky Research Medical University, Nizhny Novgorod 603082, Russia; 2 Research Institute of Clinical Oncology “Nizhny Novgorod Regional Clinical Oncological Dispensary”, Nizhny Novgorod 603093, Russia

**Keywords:** cell-in-cell, emperipolesis, adenocarcinoma, colorectal cancer, invasive front, poor prognostic factor

## Abstract

**Background**: Cell-in-cell (CIC) structure is a histological picture of a whole cell inside another cell. Homotypic CIC structures formed by cancer cells are consistently demonstrated to be a factor of poor prognosis and resistance to chemo- and immunotherapy in colorectal cancer (CRC). However, the absence of a standardized counting method limits the use of this factor in the applied research. **Objective**: To propose an adapted method for quantifying CIC structures in CRC surgical specimens and to evaluate their correlation with established adverse prognostic factors. **Methods**: A total of 250 histological slides of surgical specimens from 58 patients with pT1-pT4 colorectal adenocarcinoma were studied. Identification of tumor cells and visualization of CIC structures were performed by immunohistochemistry (CK20). Quantitative assessment was performed on digital scans of H&E stained slides. Quantitative assessment was performed on digital slide scans stained with H&E. CIC structures were counted in 5 fields of view corresponding to a ×40 objective (0.975 mm^2^). A correlation analysis of CIC structures with CRC poor prognosis factors was performed. **Results**: Immunohistochemical study (CK20) confirmed the formation and prevalence of homotypic structures (95%) over heterotypic ones (5%) (*p* < 0.001). This finding informed the evaluation of H&E-stained slides and the formulation of criteria for CIC structure identification. A significant predominance of CIC structures in the invasive front was established compared to the tumor central zone (16.7 ± 5.2 and 1.2 ± 1.3 per 5 fields of view, respectively, *p* < 0.0001). Correlation analysis revealed weak but statistically significant relationships with the tumor-stromal ratio, the tumor buds number and the density of tumor-infiltrating lymphocytes. No correlations were found with the right- or left-sided location, pTNM, grading, lymphovascular and perineural invasion. **Conclusions**: The paper presents the adapted CIC structures counting method for surgical specimens of CRC, defines the criteria of the CIC, and demonstrates a higher number of CIC structures in the tumor invasive front. Weak correlations between the CIC structures and established factors of CRC poor prognosis are obtained.

## 1. Introduction

Colorectal cancer (CRC) is the third most frequently diagnosed cancer and the second leading cause of cancer-related mortality worldwide [1]. The primary method for treating CRC remains radical surgical intervention, while histological examination plays a crucial role in diagnosis and precise tumor typing. The management strategy for patients with colorectal cancer is determined by considering the pTNM classification, AJCC staging, and a number of clinical, morphological, and molecular-genetic factors that poorly impact prognosis [2,3].

Despite advancements in staging protocols and therapeutic approaches, determination of the optimal management for CRC patients remains a complex challenge. It is widely recognized that patients with tumors of the same AJCC stage can differ both in terms of prognosis and response to therapy. The greatest difficulties in selecting the strategy for adjuvant chemotherapy arise in Stage II CRC, where a variable prognosis of survival is observed [3]. Thus, additional, stage-independent prognostic factors are necessary for risk stratification and decision-making regarding adjuvant treatment. This necessity has prompted pathologists to develop new criteria that can further refine risk stratification in CRC. Subsequently, parameters reflecting tumor budding and immune response were developed and incorporated into the WHO Classification of Tumours of the Digestive System 2019 recommendations for supplementary pathological assessment [4]. However, none of the proposed morphological criteria for assessment possess predictive significance (forecasting treatment efficacy).

In recent scientific publications, the assessment of CIC morphological structures has been proposed as a novel possible prognostic morphological factor in CRC [5]. CIC structures represent a histological pattern where one cell (the internalized cell) is located within another cell (the host cell) [6]. The formation of CIC structures can occur between two epithelial tumor cells (homotypic structures) or between different cell types (heterotypic structures). The host cell may be a tumor or stromal cell, while the internalized cell can be a tumor, stromal, or immune cell [7]. CIC structures are formed as a result of processes such as emperipolesis, entosis, and cellular cannibalism, which differ in the mechanism and fate of both the internalized and host cells [7,8].

It is important to note that the term emperipolesis was historically the first and was used for a long time in literature as a synonym for the CIC morphological pattern. However, subsequently, it began to be used solely to denote the mechanism of active penetration of immune cells (neutrophils, lymphocytes, and plasma cells) into normal and tumor cells [9].

Numerous data indicate that CIC structures have potential as both a prognostic and a predictive morphological factor. It has been established that the quantity of homotypic CIC structures significantly correlates with poor prognosis in breast cancer [10,11], adenocarcinoma and non-small cell lung cancer [12,13], pancreatic cancer [14], hepatocellular carcinoma [15], rectal cancer [16], bladder cancer [17], head and neck squamous cell carcinoma [16,18], and early-stage tongue cancer [19].

According to the CRC, prior studies demonstrated the engulfment of cytotoxic T lymphocytes by tumor cells, followed by the subsequent apoptotic death of the lymphocytes [20]. This phenomenon correlated with the tumor grade and the level of interleukin-6 (IL-6) [21]. The formation of homotypic CIC structures in CRC has been confirmed using confocal microscopy with double immunofluorescence (Cytokeratin, E-cadherin) staining [5,22], electron microscopy [23], and, more importantly, with three-dimensional confocal microscopy [22] in surgical specimens.

The CIC phenomenon may be viewed as a form of competition among tumor cells that promotes tumor progression through two mechanisms: (1) the winning cells exhibit a high frequency of cytokinesis failure, leading to aneuploidy; and (2) the winning cells acquire nutrients from the cells they engulf, enabling them to persist and proliferate [8,24]. The formation of CIC structures is not always accompanied by the death of the internalized cell, which can survive for extended periods inside the host cell. This persistence can potentially result in the division of the internalized cells and their subsequent exit from the host cell.

CIC formation can be considered a process that generates a safe environment where the internalized tumor cell can evade the effects of chemotherapeutic agents or immune surveillance [25]. It has been demonstrated that taxanes and nintedanib promote CIC formation, which increases resistance to these agents [26,27]. Furthermore, tumor xenografts with acquired oxaliplatin resistance exhibited a higher quantity of CICs than their non-resistant counterparts [28]. CIC structures also form upon exposure to anti-PD-L1 antibodies and provide a survival advantage for tumor cells under NK cell cytotoxicity [22]. It is known that mismatch repair-deficient CRC, which is highly sensitive to immune checkpoint blockade, forms few CIC structures, in contrast to pleomorphic carcinoma, which more frequently forms CICs and is resistant to immunotherapy [22], although both types of adenocarcinomas are poorly differentiated [28,29]. It has also been shown that the formation of CIC structures in colorectal adenocarcinoma, breast adenocarcinoma, and melanoma underlies immunotherapy resistance by protecting the internalized cells from T-cell lytic granules [25].

Thus, a consensus currently exists regarding the significance of CIC structures as a factor of poor prognosis and/or treatment efficacy in numerous oncological diseases. However, a uniform and standardized approach for the quantitative assessment of CIC structures is lacking, which limits the wide application of this method in translational research.

The purpose of the present study was to develop and validate the method for quantifying cell-in-cell structures in colorectal cancer surgical specimens, and to assess the correlation between the quantity of cell-in-cell structures and established factors of poor prognosis during the retrospective study.

## 2. Materials and Methods

The study was performed using the archive material provided by the Department of Pathology at the Nizhny Novgorod Regional Clinical Oncological Dispensary. The material comprised 250 histological slides of surgical specimens from 58 patients who received surgical treatment in 2021 and 2022 years due to adenocarcinoma of the colon, sigmoid, or rectum, staged pT1–pT4, who underwent treatment in 2021–2022. Totally 54 patients with adenocarcinoma of no special type (NOS) of tubuloglandular, cribriform, or villous structure, and 4 patients with mucinous adenocarcinoma were included in the study. All specimens were from patients without any neoadjuvant therapy. Data about MSI/MMR status are available only for seven patients. The clinical and morphological characteristics of the patients are presented in Table 1.

Immunohistochemical (IHC) study was performed using the Anti-E Cadherin (1:400; EP700Y; Abcam, Cambridge, UK) and Anti-Cytokeratin 20 (1:750; EPR1622Y; Abcam, Cambridge, UK) and monoclonal antibody. E-cadherin (E-cad) was used to confirm the formation of CIC structures, and Cytokeratin 20 (CK20) was used to assess the formation of homotypic and heterotypic CIC structures. Diaminobenzidine was used as the chromogen for the IHC reactions. The quantification of homotypic and heterotypic CIC structures was conducted in six representative tumor specimens the entire tumor area, specifically in five high-power fields corresponding to a ×40 objective (field of view 0.196 mm^2^).

The quantification of CIC structures on slides stained with Hematoxylin and Eosin (H&E) was performed using whole-slide images scanned with 0.243 µm/pixel resolution using a Pannoramic 250 slide scanner (3DHistech, Budapest, Hungary), equipped with a Plan-Apochromat ×20 objective with a numerical aperture of 0.8. The scanned slides were viewed using the SlideViewer 2.7 software (3DHistech, Budapest, Hungary).

Structures were quantified at the invasive front and in the tumor center, utilizing five high-power fields corresponding to a ×40 objective with a field of view of 0.196 mm^2^. The invasive front was defined as the border of the primary tumor where cancer cells actively disseminate into the surrounding healthy tissue. The tumor center was defined as an area of the primary tumor removed from the invasive front by at least two ×40 objective fields of view. During quantification, the fields of view were uniformly distributed along the invasive front or the tumor center in regions rich in tumor cells. Areas predominantly composed of the stromal component, necrosis, and mucin pools were excluded from the analysis.

The following criteria for the identification of CIC structures in H&E-stained sections were proposed (Table 2), including obligatory criteria: (1) the host cell nucleus displaced to the periphery, (2) the internalized cell nucleus without signs of apoptosis, (3) the host cell cytoplasm, which may not always have a distinct boundary with adjacent tumor cells but must surround the internalized cell on all sides, and additional criteria: (4) the internalized cell cytoplasm, (5) an intermediate vacuolar space.

For each patient, the following parameters were assessed: tumor localization (right-sided or left-sided), pTNM stage, grade, the presence of lymphovascular invasion and perineural invasion, the quantity and grade of tumor budding, the density of tumor-infiltrating lymphocytes, and the presence of a peritumoral lymphocytic reaction of the Crohn-like type in accordance with the recommendations outlined in the WHO Classification of Tumours of the Digestive System, 5th Edition (2019) [3]. The determination of the tumor-to-stroma ratio was performed according to the methodology described by van Pelt GW et al. [30].

### Statistical Methods and Data Processing

Statistical analysis and graphical visualization were performed using the GraphPad Prism 10.2.3 software (GraphPad Software, Boston, MA, USA). The level of statistical significance was calculated using the non-parametric Mann–Whitney U-test. Results were considered statistically significant if the *p*-value was less than 0.05. The correlation analysis between the quantity of CIC structures and the WHO-recommended criteria were conducted using Spearman’s rank correlation coefficient (*ρ*). The level of association between the investigated parameters was interpreted based on the obtained correlation coefficient values: weak correlation for 0.3 ≥ *ρ* < 0.5; moderate correlation for 0.5 ≥ *ρ* < 0.7; high correlation for 0.7 ≥ *ρ* < 0.9; and very high correlation for 0.9 ≥ *ρ* ≤ 1.

Intraclass Correlation Coefficients (ICC) estimates and their 95% confident intervals were calculated using SPSS statistical package version 16 (SPSS Inc, Chicago, IL, USA) based on a single-rating, absolute-agreement, 2-way mixed-effects model. Based on the 95% confident interval of the ICC estimate, values less than 0.5, between 0.5 and 0.75, between 0.75 and 0.9, and greater than 0.90 are indicative of poor, moderate, good, and excellent reliability, respectively [31].

## 3. Results

### 3.1. Feasibility of Assessing Homotypic Cell-in-Cell Structures and Their Evaluation Criteria

**Figure 1:**
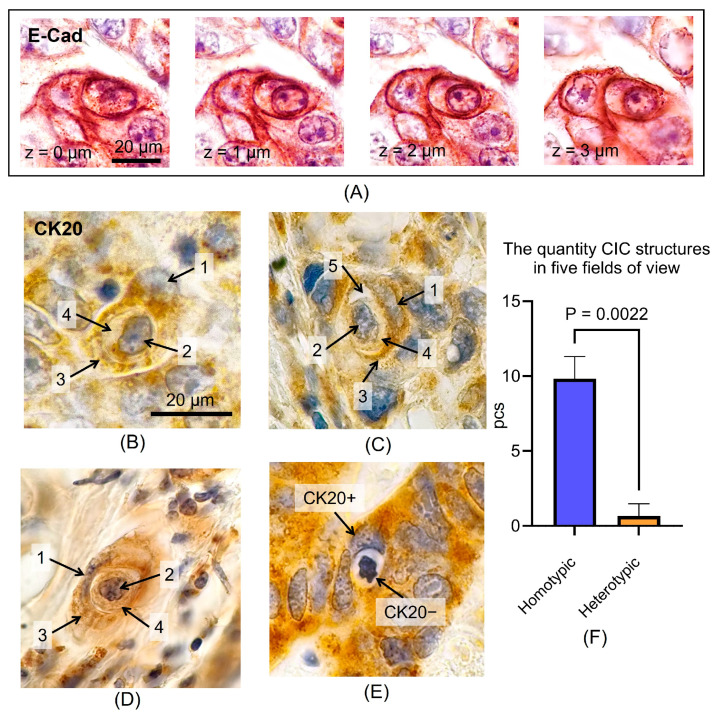
Identification of homotypic and heterotypic CIC structures using IHC of CK20. (**A**)—z-stack of the CIC structure with a step of 1 μm; (**B**–**D**)—example of homotypic CIC structures consisting of two CK20+ epithelial cancer cells; (**E**)—example of a heterotypic CIC structure consisting of a CK20+ host cell and a CK20− internalized cell; (**F**)—results of quantitative assessment of homotypic and heterotypic structures in CRC specimens; structural elements of CIC: 1—‘moon-shape’ host cell nucleus, 2—internalized cell nucleus, 3—host cell cytoplasm, 4—internalized cell cytoplasm, 5—intervening vacuolar space.

Using E-cadherin to visualize cell membranes confirmed the presence of CIC structures in adenocarcinoma samples. However, CIC structures in sections can only be detected when the section passes through the nuclei of both cells (the so-called central section of the cell), and at a specific position of the focal plane, as seen in the Z-stack in Figure 1A.

Since the formation of CIC structures can occur between two tumor cells (homotypic CIC) or between a tumor cell and a lymphocyte (heterotypic CIC) [22], an IHC reaction targeting the epithelial cell marker CK20 was performed. Structures composed of two CK20+ cells (Figure 1B–D) and structures with an internalized CK20- cell (Figure 1E) were identified. Quantitative assessment demonstrated a prevalence of homotypic structures over heterotypic structures (9.8 ± 1.5 pcs vs 0.7 ± 0.8 pcs, *p* = 0.0022). Thus, the examination of H&E-stained sections predominantly allows for the quantification of homotypic structures (Figure 1D), which is important to consider when evaluating the potential prognostic utility of this criterion.

**Figure 2:**
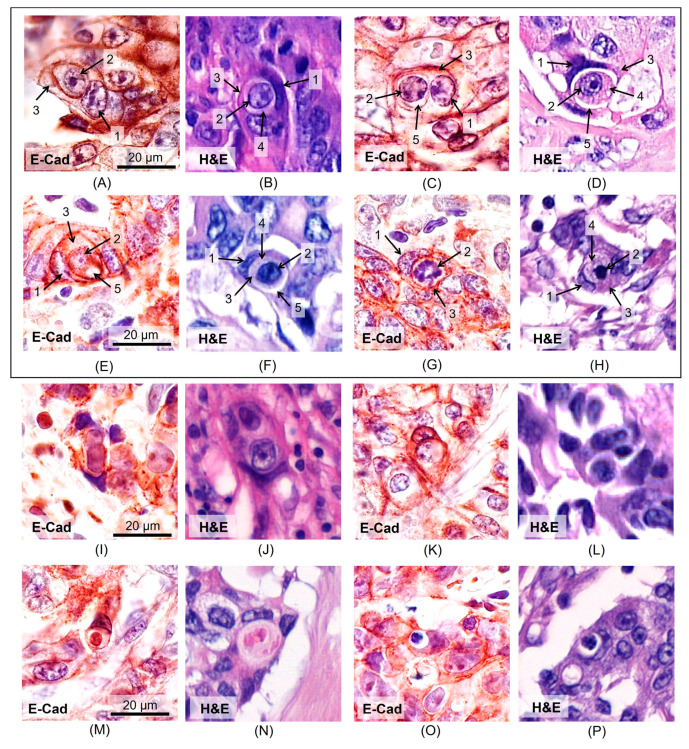
Types of the CIC structures that can be identified on scans of H&E-stained sections (**A**–**H**) and structures that do not fit the definition of CIC (**I**–P). (**A**,**B**)—CIC structure without intervening vacuolar space; (**C**–**F**)—CIC structures with an intervening vacuolar space; (**G**,**H**)—internalized cells show signs of mitosis; (**I**–**L**)—the host cell does not completely surround the other cell; (**M**,**N**)—the nucleus of the internalized cell is absent, fragments of cytoplasm may be apoptotic bodies; (**O**,**P**)—internalized cells with signs of apoptosis: karyopyknosis, karyorrhexis, hypereosinophilic cytoplasm; structural elements of CIC: 1—‘moon-shape’ host cell nucleus, 2—internalized cell nucleus, 3—host cell cytoplasm, 4—internalized cell cytoplasm, 5—intervening vacuolar space.

The CIC structures observed during IHC analysis and on H&E-stained slides were analyzed. All CIC structures included four structural elements: (1) the host cell nucleus displaced toward the periphery, which in some cases presented with a ‘moon-shape’, (2) the internalized cell nucleus, (3) the host cell cytoplasm, which circumscribes the internalized cell on all sides, and (4) the internalized cell cytoplasm (Figure 1B and Figure 2A,B). The majority of CIC structures featured an intervening vacuolar space (Figure 1C and Figure 2C–F). Upon E-cad staining, the cell cytoplasm exhibited poor contrast, yet the cell boundaries were the most evident. Conversely, with CK20 and H&E staining, the cellular membranes were less distinct (Figure 1 and Figure 2). Structures containing mitotic figures within the internalized cells were also observed among the CIC structures (Figure 2G,H). This phenomenon has been previously described [25] and does not contradict the CIC concept.

Analysis of the H&E-stained slides revealed structures that could be mistakenly identified as CICs (Pseudo-CIC). These include: densely packed cells that do not completely surround another cell or lack a distinct cellular membrane, which significantly impedes accurate evaluation (Figure 2I,J); and cells containing internalized apoptotic bodies or apoptotic cells (Figure 2M,N).

### 3.2. Localization of Cell-in-Cell Structures Within the Tumor

Previously, studies investigating the prognostic significance of CIC structures did not examine the distribution of these structures within colorectal adenocarcinoma [12,22]. However, considering that the invasive front of the tumor is in direct contact with intact tissue and is considered the location of the most aggressive cells associated with cellular invasion, acquisition of stem cell properties, therapy resistance, and immune suppression [32,33], a heterogeneous distribution of CIC structures between the invasive front and the tumor center cannot be excluded. Prognostic factors such as tumor budding, tumor-to-stroma ratio, and tumor-infiltrating lymphocytes are typically assessed at the invasive front.

**Figure 3:**
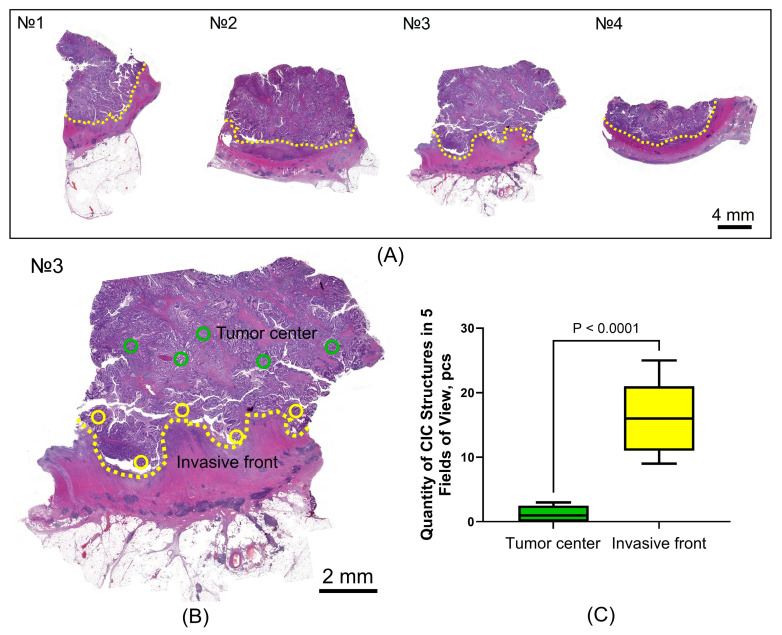
Specimens selection for analysis and subsequent evaluation of CIC structures in the center and at the invasion front of the tumor. (**A**)—Histological slides of colorectal cancer samples from one patient; the sample with the greatest depth of invasion into the colon wall was selected; (**B**)—Distribution of fields of view (0.199 mm^2^) by the tumor center and the invasion front; (**C**)—Results of the quantitative assessment of CIC structures. Green circles—fields of view at the tumor center, yellow circles—fields of view at the invasive front, yellow dotted line—invasive front.

For the visualization of components and the quantification of CIC structures, a ×40 objective is optimal [12]. In order to standardize the methodology, we calculated the area of the field of view for the most common combination of a ×40 objective and a ×10 eyepiece with a 20-field number, which amounted to 0.196 mm^2^. Quantification was performed on digitized histological slides in five fields of view (0.196 mm^2^) uniformly distributed across the invasive front and the tumor center. The result was expressed in pcs per 0.975 mm^2^. For assessment, the histological slide of adenocarcinoma with the greatest depth of invasion into the intestinal wall and/or adjacent tissues or organs was selected (Figure 3A,B).

A significantly higher distribution of CIC structures was demonstrated at the invasive front than in the tumor center, with values of 16.7 ± 5.2 pcs vs 1.2 ± 1.3 pcs, respectively (*p* < 0.0001) (Figure 3C).

### 3.3. Estimation of Inter-Rater Reliability and Correlation Analysis of Cell-in-Cell Structures with Established Poor Prognostic Factors

The quantity of CIC structures according to the proposed methodology was assessed for ten colorectal cancer samples by three experts (AP, RS, ID) following preliminary training. The ICC was 0.77 (95% CI 0.59–0.89, *p* < 0.0001), which corresponds to a reliability level ranging from “moderate” to “good”. It is worth noting that despite the experts being familiarized with the proposed assessment methodology, they made errors, quantifying structures in which the host cell did not completely surround the internalized cell, and could also miss structures in solid tumor areas with dense packing of tumor cells. Thus, this assessment requires high quality training of experts.

**Figure 4:**
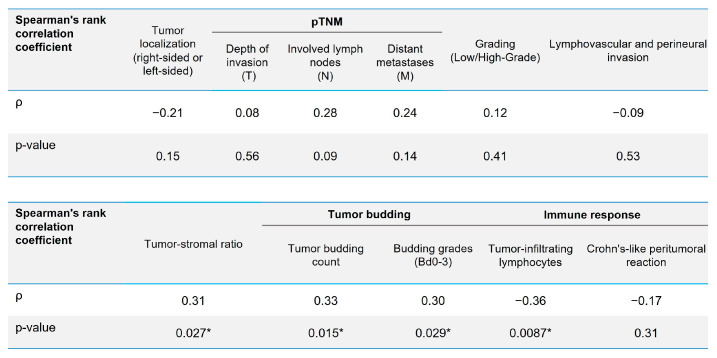
The result of the correlation analysis between the number of CRC structures in colorectal adenocarcinoma and the established poor prognostic factors. *—statistically significant correlation.

A correlation analysis was performed to evaluate the relationship between the quantity of CIC structures, considered a novel prognostic marker, and previously established poor prognostic factors. Statistically significant (*p* < 0.05), but weak (*ρ* < 0.5) correlations were demonstrated with the following parameters: tumor-to-stroma ratio, tumor budding count, grade of tumor budding, and density of tumor-infiltrating lymphocytes (Figure 4).

No statistically significant correlations were found between the quantity of CIC structures and clinicopathological parameters such as: tumor localization (left- or right-sided), depth of invasion (pT), number of involved lymph nodes (pN), distant metastases (pM), grading, lymphovascular invasion, perineural invasion, and peritumoral Crohn-like lymphocytic reaction.

These results indicate the independence of CIC structures from a number of clinicopathological parameters as a possible prognostic factor.

## 4. Discussion

In this study, the feasibility of applying the method for quantifying CIC structures in colorectal adenocarcinoma surgical samples was demonstrated. The methodology for CIC structure quantification was adapted for the first time for slides and digital whole-slide images stained with H&E, taking into account (1) the selection of the slide from the surgical samples, (2) the choice of the area of quantification within the tumor, (3) the selection of the objective field of view, and (4) the optimal criteria for CIC structures, making it suitable for translational research.

A limitation of quantifying CIC structures on routinely H&E-stained thin sections (4–6 µm) is the inability to assess the three-dimensional organization and formation of the structures, exclude overlays artifact, and selectively quantify homotypic versus heterotypic structures. Although the existence of homotypic CIC structures composed of cancer cells has been confirmed in numerous scientific studies [22,23,28], the objectivity of quantification on sections compared to three-dimensional visualization has not been assessed.

Previous studies have shown that the use of H&E staining is sufficient for the quantitative assessment of CIC structures and does not introduce a significant error compared to IHC reaction to E-cadherin, which allows for the visualization of cell membrane boundaries [14,19]. The use of IHC reaction to CK20 in our study allowed us to establish the formation and multi-fold prevalence of homotypic CIC structures over heterotypic structures, which is consistent with the data reported by Lin YY et al. and Bozkurt E et al. [21,22] for CRC samples.

Previously, the method for quantifying CIC structures in H&E-stained histological slides was proposed by Mackay et al. [12]. However, this method exhibits several significant drawbacks. According to this protocol, CIC structures must include a minimum of four out of six components: the internalized cell nucleus, the internalized cell cytoplasm, the host cell nucleus, the crescent-shaped host cell nucleus, the host cell cytoplasm, and the intermediate vacuolar space. However, the presence of just four components, as per the Mackay et al. protocol, may not always genuinely correspond to a true CIC structure. For example, a structure that does not include the internalized cell nucleus is visually indistinguishable from internalized apoptotic bodies. The criteria proposed in the present study eliminate such potential inaccuracies.

The Mackay et al. method suggests that quantification should be performed in viable, epithelial cell-rich tumor areas and does not account for the distribution of structures at the invasive front of the tumor. We previously demonstrated that in colorectal cancer xenografts in mice (HT29 and HCT116 lines), a greater number of CIC structures were located at the invasive front of the tumor than in the central zone [28]. In the current work, we confirmed this result in human colorectal adenocarcinoma samples. Since it is known that cancer stem cells play a critical role in CIC formation in CRC [22], and they are more frequently found at the invasive front [34], this may partially explain why CICs are heterogeneously distributed. Additionally, the loss of cancer cell adhesion resulting from epithelial-mesenchymal transition (EMT) occurs at the invasive front [35], which has been proven to lead to the formation of CIC structures [36]. An increase in CIC structures relative to the tumor center has also been described for non-small cell lung cancer (NSCLC) [16], suggesting a possible universality of this phenomenon.

The assessment of the correlation between the formation of CIC structures and other frequently evaluated morphological factors of prognosis showed a weak, but statistically significant correlation with the tumor-to-stroma ratio, tumor budding status, and tumor-infiltrating lymphocytes. So, we obtained the preliminary evidence that CIC density correlates weakly with established adverse markers, possibly reflecting a distinct biological process.

A limitation of this study is the homogeneous nature of the sample, as the majority of samples were classified as adenocarcinoma NOS, with only four samples represented by mucinous adenocarcinoma. Other histological subtypes characterized by a poor prognosis were absent (Table 1). Also, it was a single-center exploratory retrospective series that requires outcome-based studies to confirm prognostic value.

Several cellular mechanisms that result in the CIC morphological pattern have been described in cancerous tumors, specifically emperipolesis, entosis, and cellular cannibalism. These processes should not be confused with phagocytosis, in which the ingestion of dead, dying (apoptotic) cells or their components, as well as living cells, is possible in cancerous tumors with the involvement of professional phagocytes (macrophages, neutrophils, monocytes, dendritic cells) [37]. In phagocytosis, the internalized structures undergo lysosomal digestion, although phagocytosis of viable cancer cells is possible. However, most human tumors overexpress anti-phagocytic proteins (CD47, PD-L1, B2M) [38], which minimizes the contribution of phagocytosis to the formation of CIC morphological structures.

The formation of CIC structures in cancerous tumors is possible either through the active engulfment by the host cell (cellular cannibalism) or through the active invasion of one cell into another (entosis, emperipolesis) [9]. Cellular cannibalism is the active ingestion process of other cancer cells or other cells from the microenvironment, such as immune cells, by cancer cells, followed by lysosomal digestion. In contrast to phagocytosis, cancer cells utilize cannibalism for nutrition, while macrophages primarily perform a scavenging function for the elimination of debris and microorganisms [37]. Entosis is characterized by the active penetration of a viable cancer cell into a neighboring cell of the same type with the involvement of adhesion molecules. Entosis can conclude either with the death of the internalized cell or with the prolonged survival of one cell inside the other, and even the subsequent exit of the internalized cell [39]. Both cellular cannibalism and entosis are known to be induced by nutrient deprivation and share molecular pathways with the process of autophagy, which is essential for cell survival under stress conditions by breaking down non-essential cellular components for energy [7]. Emperipolesis is used as a term to denote the process of cell entry, movement within, and exit from other cells. This phenomenon is characteristic of immune cells (neutrophils, lymphocytes, plasma cells) that penetrate megakaryocytes, endothelial cells, and cancer cells, thereby forming heterotypic CICs. The fate of the internalized cells is also heterogeneous, as cells may undergo mitosis, exit the host cells, or die via apoptosis [7].

While the terms emperipolesis, entosis, and cellular cannibalism have occasionally been used in the morphological analysis of clinical biopsy specimens, we adhere to the perspective of Overholtzer M. et al. that these terms should be used only when the specific mechanism of CIC structure formation is known and described [6].

Since the quantification of CIC structures is laborious and increases the working time of the pathologist, our future research will be focused on utilizing machine learning and artificial intelligence methods for the automated analysis of whole-slide histological scans. It should be mentioned that artificial intelligence has already made significant progress in CRC image analysis, in histopathology, algorithms based on deep learning have the potential to assist in diagnosis, predict clinically relevant consensus molecular subtypes and molecular spatial heterogeneity [40], microsatellite instability, identify histological features related to prognosis and correlated to metastasis, and assess the specific components of the tumor microenvironment [41]. Such algorithms analyzing H&E and IHC slides. In the context of this study the Custom Convolutional Neural Networks, which are able to detect and classify nuclei and evaluate tumor budding are the closest prototypes of potential architecture for the automatic recognizing of CIC structures in H&E slides [42].

## 5. Conclusions

This study presents a methodology for the quantification of CIC structures on H&E-stained histological slides, utilizing digital scans of colorectal cancer surgical specimens. We defined the obligatory and supplementary criteria for the identification of CIC structures, specified the area of quantification within the tumor, and determined the optimal field of view.

IHC staining for CK20 demonstrated a significant predominance of homotypic CIC structures, formed by tumor cells, relative to heterotypic structures. We report the first finding of a greater number of CIC structures forming at the invasive front compared to the central tumor area of adenocarcinoma (*p* < 0.0001) in clinical CRC material, a factor that is essential to consider during the quantitative analysis of CIC structures.

The observed weak (*ρ* = 0.30–0.36), yet statistically significant correlations (*p* < 0.05) between the quantity of CIC structures and specific clinicopathological parameters indicate the independent prognostic value of the CIC index.

For CIC to be implemented as a morphological criterion in clinical practice, further validation is required to confirm the association between the assessment of H&E-stained sections and the objective three-dimensional visualization of CIC formation in surgical specimens. Furthermore, large-scale studies are necessary to establish the prognostic and predictive significance of CIC structures in human CRC.

## Figures and Tables

**Table 1:** Clinical and morphological characteristics of patients with colorectal adenocarcinoma.

	pT1	pT2	pT3	pT4
Number of patients	5	8	34	11
Mean age (range), years	63 (58–67)	67 (62–79)	69 (56–83)	68 (58–82)
Sex (Male:Female)	3:2	2:6	20:14	6:5
Lymph node involvement:				
pN0	5	8	16	4
pN1a	0	0	9	4
pN1b	0	0	5	2
pN1c	0	0	2	0
pN2a	0	0	2	1
pN2b	0	0	0	0
Distant metastases:				
pM0	5	8	33	7
pM1a	0	0	2	4
Histological subtype:				
Adenocarcinoma NOS	5	8	32	9
Mucinous adenocarcinoma ^1^	0	0	2	2

^1^ Histological subtype of CRC, which are poor prognostic factors according to WHO, signet ring cell and medullary adenocarcinoma were absent in the sampling [3].

**Table 2:** Morphological structures that must be clearly visualized to determine the CIC structures and pseudo-CIC structures.

Criteria	CIC Structures	Pseudo-CIC Structures
The host cell nucleus displaced to the periphery	+	±
The internalized cell nucleus	+	±
The host cell cytoplasm, which circumscribes the internalized cell on all sides	+	±
The internalized cell cytoplasm	±	±
An intermediate vacuolar space	±	±
Internalized apoptotic bodies or signs of apoptosis in internalized cell	-	±

## Data Availability

The original contributions presented in this study are included in the article. Further inquiries can be directed to the corresponding author.

## Abbreviations

The following abbreviations are used in this manuscript:

CIC	Cell-in-cell
CRC	Colorectal cancer
CK20	Cytokeratin-20
E-cad	E-cadherin
pTNM	Pathology TNM
AJCC	American Joint Committee on Cancer
WHO	World Health Organization
NOS	No special type
IHC	Immunohistochemistry
H&E	Hematoxylin and eosin
ICC	Intraclass Correlation Coefficients

## References

[1] Bray F., Laversanne M., Sung H., Ferlay J., Siegel R.L., Soerjomataram I., Jemal A. (2024). Global Cancer Statistics 2022: GLOBOCAN Estimates of Incidence and Mortality Worldwide for 36 Cancers in 185 Countries. CA Cancer J. Clin..

[2] Chen K., Collins G., Wang H., Toh J.W.T. (2021). Pathological Features and Prognostication in Colorectal Cancer. Curr. Oncol..

[3] Nagtegaal I.D., Odze R.D., Klimstra D., Paradis V., Rugge M., Schirmacher P., Washington K.M., Carneiro F., Cree I.A. (2020). The 2019 WHO Classification of Tumours of the Digestive System. Histopathology.

[4] Hashiguchi Y., Muro K., Saito Y., Ito Y., Ajioka Y., Hamaguchi T., Hasegawa K., Hotta K., Ishida H., Ishiguro M. (2020). Japanese Society for Cancer of the Colon and Rectum (JSCCR) Guidelines 2019 for the Treatment of Colorectal Cancer. Int. J. Clin. Oncol..

[5] Gottwald D., Putz F., Hohmann N., Büttner-Herold M., Hecht M., Fietkau R., Distel L. (2020). Role of Tumor Cell Senescence in Non-Professional Phagocytosis and Cell-in-Cell Structure Formation. BMC Mol. Cell Biol..

[6] Overholtzer M., Brugge J.S. (2008). The Cell Biology of Cell-in-Cell Structures. Nat. Rev. Mol. Cell Biol..

[7] Fais S., Overholtzer M. (2018). Cell-in-Cell Phenomena, Cannibalism, and Autophagy: Is There a Relationship?. Cell Death Dis..

[8] Druzhkova I., Ignatova N., Shirmanova M. (2023). Cell-in-Cell Structures in Gastrointestinal Tumors: Biological Relevance and Clinical Applications. J. Pers. Med..

[9] Fais S., Overholtzer M. (2018). Cell-in-Cell Phenomena in Cancer. Nat. Rev. Cancer.

[10] Dziuba I., Gawel A.M., Tyrna P., Machtyl J., Olszanecka M., Pawlik A., Wójcik C., Bialy L.P., Mlynarczuk-Bialy I. (2023). Homotypic Entosis as a Potential Novel Diagnostic Marker in Breast Cancer. Int. J. Mol. Sci..

[11] Ruan B., Niu Z., Jiang X., Li Z., Tai Y., Huang H., Sun Q. (2019). High Frequency of Cell-in-Cell Formation in Heterogeneous Human Breast Cancer Tissue in a Patient with Poor Prognosis: A Case Report and Literature Review. Front. Oncol..

[12] Mackay H.L., Moore D., Hall C., Birkbak N.J., Jamal-Hanjani M., Karim S.A., Phatak V.M., Piñon L., Morton J.P., Swanton C. (2018). Genomic Instability in Mutant P53 Cancer Cells upon Entotic Engulfment. Nat. Commun..

[13] Liu X., Guo R., Li D., Wang Y., Ning J., Yang S., Yang J. (2024). Homotypic Cell-in-Cell Structure as a Novel Prognostic Predictor in Non-Small Cell Lung Cancer and Frequently Localized at the Invasive Front. Sci. Rep..

[14] Hayashi A., Yavas A., McIntyre C.A., Ho Y., Erakky A., Wong W., Varghese A.M., Melchor J.P., Overholtzer M., O’Reilly E.M. (2020). Genetic and Clinical Correlates of Entosis in Pancreatic Ductal Adenocarcinoma. Mod. Pathol..

[15] Wang R., Zhu Y., Zhong H., Gao X., Sun Q., He M. (2022). Homotypic Cell-in-Cell Structures as an Adverse Prognostic Predictor of Hepatocellular Carcinoma. Front. Oncol..

[16] Schwegler M., Wirsing A.M., Schenker H.M., Ott L., Ries J.M., Büttner-Herold M., Fietkau R., Putz F., Distel L.V. (2015). Prognostic Value of Homotypic Cell Internalization by Nonprofessional Phagocytic Cancer Cells. BioMed Res. Int..

[17] Huang H., Sun R., Yang J. (2021). The Value of cellular devouring in Cytopathological Diagnosis. Clin. Exp. Pathol..

[18] Schenker H., Büttner-Herold M., Fietkau R., Distel L.V. (2017). Cell-in-Cell Structures Are More Potent Predictors of Outcome than Senescence or Apoptosis in Head and Neck Squamous Cell Carcinomas. Radiat. Oncol..

[19] Almangush A., Mäkitie A.A., Hagström J., Haglund C., Kowalski L.P., Nieminen P., Coletta R.D., Salo T., Leivo I. (2020). Cell-in-Cell Phenomenon Associates with Aggressive Characteristics and Cancer-Related Mortality in Early Oral Tongue Cancer. BMC Cancer.

[20] Salvesen G.S. (2014). Dying from within: Granzyme B Converts Entosis to Emperitosis. Cell Death Differ..

[21] Bozkurt E., Kisakol B., Azimi M., Dussmann H., Cho S., McDonough E., Fay J., O’Grady T., Burke J.P., McCawley N. (2025). Multiplex Analysis of Colorectal Cancer Tissue Describes the Composition, Cell Biology and Spatial Effects of Cell-in-Cell Events and Identifies a T Cell-Dependent Prognostic Signature. bioRxiv.

[22] Lin Y.-Y., Lan H.-Y., Teng H.-W., Wang Y.-P., Lin W.-C., Hwang W.-L. (2025). Colorectal Cancer Stem Cells Develop NK Cell Resistance via Homotypic Cell-in-Cell Structures Suppressed by Stathmin1. Theranostics.

[23] Bozkurt E., Düssmann H., Salvucci M., Cavanagh B.L., Van Schaeybroeck S., Longley D.B., Martin S.J., Prehn J.H.M. (2021). TRAIL Signaling Promotes Entosis in Colorectal Cancer. J. Cell Biol..

[24] Florey O., Kim S., Overholtzer M. (2015). Entosis: Cell-in-Cell Formation That Kills Through Entotic Cell Death. Curr. Mol. Med..

[25] Gutwillig A., Santana-Magal N., Farhat-Younis L., Rasoulouniriana D., Madi A., Luxenburg C., Cohen J., Padmanabhan K., Shomron N., Shapira G. (2022). Transient Cell-in-Cell Formation Underlies Tumor Relapse and Resistance to Immunotherapy. eLife.

[26] Liu J., Wang L., Zhang Y., Li S., Sun F., Wang G., Yang T., Wei D., Guo L., Xiao H. (2019). Induction of Entosis in Prostate Cancer Cells by Nintedanib and Its Therapeutic Implications. Oncol. Lett..

[27] Durgan J., Tseng Y.-Y., Hamann J.C., Domart M.-C., Collinson L., Hall A., Overholtzer M., Florey O. (2017). Mitosis Can Drive Cell Cannibalism through Entosis. eLife.

[28] Druzhkova I., Potapov A., Ignatova N., Bugrova M., Shchechkin I., Lukina M., Shimolina L., Kolesnikova E., Shirmanova M., Zagaynova E. (2024). Cell Hiding in Colorectal Cancer: Correlation with Response to Chemotherapy in Vitro and in Vivo. Sci. Rep..

[29] Mei W.-J., Mi M., Qian J., Xiao N., Yuan Y., Ding P.-R. (2022). Clinicopathological Characteristics of High Microsatellite Instability/Mismatch Repair-Deficient Colorectal Cancer: A Narrative Review. Front. Immunol..

[30] van Pelt G.W., Sandberg T.P., Morreau H., Gelderblom H., van Krieken J.H.J.M., Tollenaar R.A.E.M., Mesker W.E. (2018). The Tumour–Stroma Ratio in Colon Cancer: The Biological Role and Its Prognostic Impact. Histopathology.

[31] Koo T.K., Li M.Y. (2016). A Guideline of Selecting and Reporting Intraclass Correlation Coefficients for Reliability Research. J. Chiropr. Med..

[32] Karamitopoulou E., Zlobec I., Koelzer V.H., Langer R., Dawson H., Lugli A. (2015). Tumour Border Configuration in Colorectal Cancer: Proposal for an Alternative Scoring System Based on the Percentage of Infiltrating Margin. Histopathology.

[33] Bryne M. (1998). The Invasive Front of Carcinomas. The Most Important Area for Tumour Prognosis?. Anticancer Res..

[34] Baker A.-M., Graham T.A., Elia G., Wright N.A., Rodriguez-Justo M. (2015). Characterization of LGR5 Stem Cells in Colorectal Adenomas and Carcinomas. Sci. Rep..

[35] Nie F., Sun X., Sun J., Zhang J., Wang Y. (2025). Epithelial-Mesenchymal Transition in Colorectal Cancer Metastasis and Progression: Molecular Mechanisms and Therapeutic Strategies. Cell Death Discov..

[36] Overholtzer M., Mailleux A.A., Mouneimne G., Normand G., Schnitt S.J., King R.W., Cibas E.S., Brugge J.S. (2007). A Nonapoptotic Cell Death Process, Entosis, That Occurs by Cell-in-Cell Invasion. Cell.

[37] Zhou L., Fan S., Zhang W., Gong Z., Wang D., Tang D. (2025). The Battle within: Cell Death by Phagocytosis in Cancer. Clin. Transl. Oncol..

[38] Brown G.C., Neher J.J. (2012). Eaten Alive! Cell Death by Primary Phagocytosis: ‘Phagoptosis’. Trends Biochem. Sci..

[39] Gaptulbarova K.A., Tsydenova I.A., Dolgasheva D.S., Kravtsova E.A., Ibragimova M.K., Vtorushin S.V., Litviakov N.V. (2024). Mechanisms and Significance of Entosis for Tumour Growth and Progression. Cell Death Discov..

[40] Le Douget J.E., Jacob P., Lepage C., Gallois C., Sroussi M., De Reynies A., Cazelles A., Gonzalez D., Maussion C., Bouche O. (2025). Deep Learning on Histologic Slides Accurately Predicts Consensus Molecular Subtypes and Spatial Heterogeneity in Colon Cancer. Mod. Pathol..

[41] Davri A., Birbas E., Kanavos T., Ntritsos G., Giannakeas N., Tzallas A.T., Batistatou A. (2022). Deep Learning on Histopathological Images for Colorectal Cancer Diagnosis: A Systematic Review. Diagnostics.

[42] Liu S., Zhang Y., Ju Y., Li Y., Kang X., Yang X., Niu T., Xing X., Lu Y. (2021). Establishment and Clinical Application of an Artificial Intelligence Diagnostic Platform for Identifying Rectal Cancer Tumor Budding. Front. Oncol..

